# Predicting Student Performance and Deficiency in Mastering Knowledge Points in MOOCs Using Multi-Task Learning

**DOI:** 10.3390/e21121216

**Published:** 2019-12-12

**Authors:** Shaojie Qu, Kan Li, Bo Wu, Xuri Zhang, Kaihao Zhu

**Affiliations:** 1 Network Information Technology Center, Beijing Institute of Technology, Beijing 100081, China; qushaojie@bit.edu.cn (S.Q.); wubo@bit.edu.cn (B.W.); 2 School of Computer Science and Technology, Beijing Institute of Technology, Beijing 100081, China; 3220180769@bit.edu.cn (X.Z.); 3220180779@bit.edu.cn (K.Z.)

**Keywords:** multi-task, multi-layer LSTM, attention mechanism, MOOCs, educational data mining

## Abstract

Massive open online courses (MOOCs), which have been deemed a revolutionary teaching mode, are increasingly being used in higher education. However, there remain deficiencies in understanding the relationship between online behavior of students and their performance, and in verifying how well a student comprehends learning material. Therefore, we propose a method for predicting student performance and mastery of knowledge points in MOOCs based on assignment-related online behavior; this allows for those providing academic support to intervene and improve learning outcomes of students facing difficulties. The proposed method was developed while using data from 1528 participants in a C Programming course, from which we extracted assignment-related features. We first applied a multi-task multi-layer long short-term memory-based student performance predicting method with cross-entropy as the loss function to predict students’ overall performance and mastery of each knowledge point. Our method incorporates the attention mechanism, which might better reflect students’ learning behavior and performance. Our method achieves an accuracy of 92.52% for predicting students’ performance and a recall rate of 94.68%. Students’ actions, such as submission times and plagiarism, were related to their performance in the MOOC, and the results demonstrate that our method predicts the overall performance and knowledge points that students cannot master well.

## 1. Introduction

Massive open online courses (MOOCs) are being increasingly used in higher education. When compared with traditional classes, MOOCs can provide many teaching resources with rich content [1], including online discussion forums, interactive simulations, and so on [2]. Furthermore, students can access MOOCs whenever and wherever they want, which can enhance students’ learning interest and initiative [3,4,5]. 

Previous researchers have demonstrated that MOOCs have positive effects on teaching and learning [6]. However, the mode makes instructors unable to easily perceive the status of students’ learning different from face-to-face interaction of traditional teaching mode. Currently, instructors check the completion of assignments or examinations [7], or the existence and extent of procrastination behavior [8] of students to predict student performance. However, assignment or examination completion and procrastination behavior do not fully reflect students’ learning achievement [9]. 

The learning process is also an important factor in influencing student performance. Therefore, we fully consider the temporal features in the learning process and present a study that is aimed at predicting overall performance and student deficiency in mastering knowledge points based on certain assignment-related online behavior in MOOCs. We extract assignment behavior-related features, which include temporal individual features that are related to individual assignments, temporal grouping features related to grouped assignments of certain knowledge point, and comprehensive features that are related to all assignments. We propose a model that is based on multi-task learning and long short-term memory (LSTM). In addition, we construct a main task for predicting students’ performance and an auxiliary task to predict students’ mastery of knowledge points.

The contributions of this paper are as follows:

- We propose a multi-task learning framework that is based on a multi-layer LSTM network to predict whether the students will pass the final exam as well as their mastery of knowledge points. Our framework combined multi-task prediction and comprehensive prediction with attention mechanism, which can mix multi-dimension knowledge and improve the prediction accuracy.
- We present an attention-based multi task LSTM neural network method that incorporates a shared parameters layer. The multi task architecture with shared parameters can avoid overfitting and improve the prediction. The LSTM neural network with attention mechanism adjusts the importance of temporal individual features, temporal grouping features, and it could reflect students’ learning behaviors well.
- The results demonstrate that our method is superior to other existing multi-task-based LSTM methods. Our framework can predict students’ performance with 92.52% accuracy and students’ mastery of knowledge points with 99.58% accuracy on the experimental dataset.

The following sections are organized, as follows: The related work is described in Section 2. In Section 3, we propose the framework and method. In Section 4, we present the data, results, and discussion of our experiments. Finally, conclusions are drawn and future research is discussed.

## 2. Related Work

### 2.1. Prediction in MOOCs

The use of MOOCs can improve students’ academic performance, and researchers have turned their attention to this field [10,11,12]. Conijn, Van, and Cuijpers [13] investigated the relationship among students’ final exam grade, activity frequencies, specific course item frequencies, and the order of activities. They demonstrated that students who spend more time on learning perform better, but no convincing correlation was found between the sequence of activities and student performance. Meier, Xu, Atan, and van der Schaar [14] proposed a method for improving the teaching effects of traditional classes and MOOCs. They suggest that assessment methods, such as MOOC quizzes, could provide a timely prediction of students’ performance, allowing for appropriate interventions to be made by instructors.

Studying different student behavior patterns can lead to improvements in the effects of teaching. Kahan, Soffer, and Nachmias [15] identified seven participant patterns, including Tasters, Downloaders, Disengagers, Offline Engagers, Online Engagers, Moderately Social Engagers, and Social Engagers, to support the idea that different behavior in MOOCs affects the certification rate. Moreover, Rodrigues, Ramos, Silva, and Gomes [16] identified the participation patterns of students in MOOCs by using educational data mining technologies. Their analysis guided the design of adaptive strategies and helped to improve the learning experience. Furthermore, Brinton, Buccapatnam, Chiang, and Poor [17] studied video viewing behavior and tested the performance among middle school students in MOOCs. They found that some of these behaviors were significantly associated with the accuracy of first-attempt responses to test questions.

In terms of cheating in MOOCs, some students cheat because MOOCs are online and it is difficult to detect cheating in online environments. Northcutt, Ho, and Chuang [18] described a spoofing strategy that used a Copying Answers while using Multiple Existences Online (CAMEO) strategy to replicate the answer using a ‘harvester’ account to provide a solution to an evaluation problem and then submit the correct answer while using a separate ‘master’ account. Alexandron, Ruipérez, Chen, Muñoz, and Pritchard [19] demonstrated academic dishonesty involving the use of multiple accounts in MOOCs to obtain authorization solutions. In their study, they were able to determine the prevalence of CAMEO, study its detailed characteristics, and infer the motivation for using it.

### 2.2. Multitask Learning

Multi-task learning is a method of machine learning that uses the shared network to carry out multiple tasks at the same time to achieve a better learning effect. Multi-task learning is widely used in biology, education, and machine learning. The two most commonly used ways to perform multi-task learning are typically hard or soft parameter sharing of hidden layers [20]. Hard parameter sharing is the most commonly used approach for multi-task learning; it shares the hidden layers between all of the tasks while keeping several task-specific output layers. Hard parameter sharing can greatly reduce the risk of overfitting. Baxter [21] demonstrated that the risk of shared parameter overfitting is order *N*, where *N* is the number of tasks, when compared with the traditional deep learning method. For soft parameter sharing, each task has its own model with its own parameters. The distance between the parameters is regularized to make the parameters more similar [22,23]. 

In recent work, researchers have paid more attention to joint learning, weighting losses, and learning with auxiliary tasks. For joint learning, Hashimoto, Xiong, Tsuruoka, and Socher [24] proposed a hierarchical architecture that consisted of several natural language processing (NLP) tasks. For the weighting losses, Kendall, Gal, and Cipolla [25] took the approach of adjusting each task’s relative weight in the loss function based on maximizing the Gaussian likelihood with task-dependent uncertainty. Regarding auxiliary tasks, there remains the need to find suitable auxiliary tasks to improve the performance of multi-Task learning. Zhang, Luo, Loy, and Tang [26] used head pose estimation and facial attribute inference as the auxiliary tasks for facial landmark detection.

Multi-Task learning is a method for improving the generalization effect by using the domain information that is contained in training signals of related tasks as inductive bias. Multi-Task learning can improve the generalization effect by learning tasks in parallel [27] while using a shared representation. 

### 2.3. LSTM

A LSTM neural network [28] is an improved recurrent neural network. It solves the problem of gradient explosion and gradient disappearance of a recurrent neural network, and it can transmit information in a long sequence. LSTM can control the transmission, storage, and forgetting of information through an input gate, output gate, and forgetting gate [29]. LSTM is widely used in action recognition [30], semantic analysis [31], and trajectory prediction [32,33]. Alahi [32] proposed a LSTM model to learn human movements and predict their future trajectories. In contrast with traditional approaches, they demonstrated that their method outperforms state-of-the-art methods on several public datasets. They analyzed the trajectories predicted to demonstrate motion behavior.

Song [30] proposed a spatial and temporal attention model for exploring features for human action recognition and detection. They built a recurrent neural network with long short-term memory units and focused on the discriminative joints of skeletons. The experimental results demonstrate that their proposed model is effective for both action recognition and action detection.

## 3. Method 

### 3.1. Framework 

This paper proposes a multi-task-based model for predicting students’ performance and mastery of knowledge points. We extract the temporal features, including temporal individual features, temporal grouping features, and comprehensive features. Temporal individual features are extracted from all of the assignments as a sequence that reflects the learning processes of students. Temporal grouping features are a sequence extracted from specific assignments related to certain knowledge points, and they reflect students’ mastery of specific knowledge points. Comprehensive features are vectors extracted from all the assignments to reflect the overall learning state of students. Thus, we use temporal individual features to predict students’ performance in the main task, temporal grouping features to predict students’ mastery of knowledge points in auxiliary tasks, and comprehensive features to predict students’ overall performance. We adjust the importance among temporal individual features, temporal grouping features, and comprehensive features while using an attention mechanism to improve the prediction performance. 

**Figure 1:**
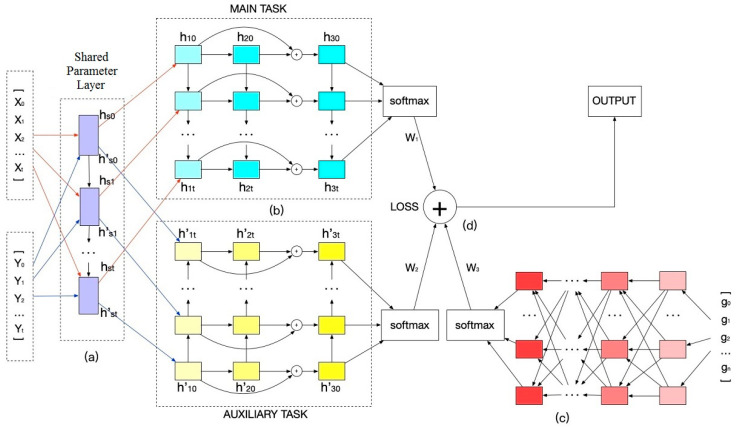
Proposed framework. (**a**) Shared parameters layer, (**b**) multi-task part with multi-layer LSTM. (**c**) multi-layer perceptron (MLP) using comprehensive features, and (**d**) attention mechanism.

This framework includes four parts, i.e., shared-parameters layer, multi-task prediction, comprehensive prediction, and attention mechanism, for predicting students’ performance and mastery of knowledge points, as shown in Figure 1. [*X*_0_, *X*_1_, *_…_*, *X_t_*] is the temporal individual feature sequence, *X_k_* is a vector extracted from assignments completed in the *k*th week, and *X_k_* is also the *k*th input of the shared parameters layer for the main task. [*Y*_0_, *Y*_1_, *_….._*, *Y_t_*] is the temporal grouping feature sequence, *Y_k_* is a vector extracted from assignments completed in the *k*th week related to specific knowledge points, and *Y_k_* is also the *k*th input of the shared parameters layer for auxiliary task. 

In Figure 1a, a shared parameters layer is then placed, and the two feature sequences share the same parameters when they are trained in the shared parameters layer. The shared parameters layer help to discover hidden information and avoid over-fitting. 

The multi-task module in Figure 1b includes the main task of predicting students’ performance and the auxiliary task of predicting students’ mastery of knowledge points. The multi-task method can mix multi-dimension knowledge and use the idea of transfer learning to improve the prediction accuracy.

In Figure 1c, we add an MLP model to focus on comprehensive features for comprehensive prediction. Here, *g* is the comprehensive feature vector that is extracted from all of the assignments completed by the student. It helps to improve the accuracy of prediction by adding comprehensive information that cannot be reflected by temporal features.

The combined loss from the MLP and multi-task with attention mechanism that is shown in Figure 1d could utilize the effective information in the temporal individual features, temporal grouping features, and comprehensive features. Attention mechanism will pay greater attention to important information, give higher weight, and improve the accuracy of prediction.

### 3.2. Multi-Task Learning

In this part, we propose a multi-task learning method that includes a main and auxiliary task to predict students’ performance and mastery of knowledge points. In the main task, we predict the students who will fail the final exam. In the auxiliary task, we predict students’ mastery of knowledge points. The main and auxiliary task both have the same architectures.

#### 3.2.1. Fully-Connected Multi-Layer LSTM Neural Network

We construct a fully-connected multi-layer LSTM neural network in the main and auxiliary tasks. It helps to enhance the importance of critical information. The input of each layer comes not only from the output of the upper layer, but also from the weighted input of all the previous layers, unlike a traditional multi-layer LSTM neural network.

Both tasks are fully-connected neural networks. For the traditional multi-layer LSTM neural network, as shown in Formula (1), the input *I_nt_* of the next layer is the output *h_n-1,t_* of the former layer, while *n* is the layer number and *t* is LSTM unit number.

(1) $$I_{nt}=h_{n-1,t}$$

For our fully-connected multi-layer LSTM, the input of the first layer of the LSTM comes from the shared parameters layer, the input *I*_2,*t*_ of the second layer comes from the output *h*_1,*t*_ of the first layer, and the input *I*_3.*t*_ of the third layer comes from the outputs *h*_1,*t*_ of the first and *h*_2,*t*_ of the second layers. The input of each layer comes from the weighted sum of the output of all the previous layers, as shown in Formula (2).

(2) $$I_{nt}=\overset{n-1}{\underset{i=1}{\sum }}W_{i}h_{i,t}$$

#### 3.2.2. Shared Parameters Layer

The shared parameters layer is a traditional LSTM neural network, and it is built, such that the inputs *X* and *Y* share the same network parameters (see Figure 1a). Subsequent to training by the shared parameters layer, the outputs of the LSTM neural network are used as the inputs of the main and auxiliary tasks. The shared parameters layer can avoid over-fitting and mix information regarding multiple tasks for improving the accuracy of prediction.

For the input *X*, *h_sk_* is the output of the feature sequence in the *k*th LSTM unit of the shared parameters layer, and it is also the *k*th input of the main task. For the input *Y*, *h’_sk_* is the output of the feature sequence in the *k*th LSTM unit of the shared parameters layer, and it is also the *k*th input of the auxiliary task. The outputs *h_sk_* and *h’_sk_* from the shared parameters layer with the input sequence *X_k_* and *Y_k_* will be the inputs of the main and auxiliary tasks, respectively. 

**Figure 2:**
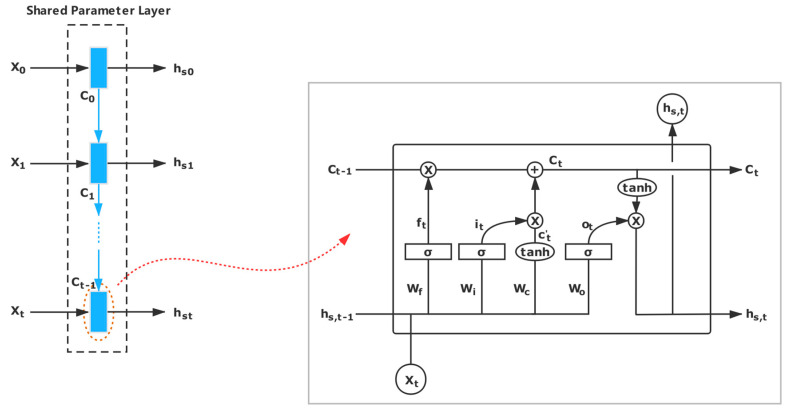
Shared parameters layer. For the inputs *X* and *Y*, they share the same network parameters by the shared parameters layer. In this figure, we take the input *X* as example.

Figure 2 shows the shared parameters layer, and we take the input *X* as an example. 

For the input *X*, the output *h_t_* is calculated while using the following equations.

(3) $$f_{t}=\sigma \left(W_{f}\left[h_{s,t-1},x_{t}\right]+b_{f}\right)$$

(4) $$\;i_{t}=\sigma \left(W_{i}\left[h_{s,t-1},x_{t}\right]+b_{i}\right)\;$$

(5) $$\;{\bar{C}}_{t}=\tanh\left(W_{c}\left[h_{s,t-1},x_{t}\right]+b_{c}\right)\;$$

(6) $$\;C_{t}=f_{t}\circ C_{t-1}+i_{t}\circ \bar{C_{t}}\;$$

(7) $$\;o_{t}=\sigma \left(W_{o}\left[h_{s,t-1},x_{t}\right]+b_{o}\right)\;$$

(8) $$\;C_{t}=\tanh\left(C_{t}\right)\;$$

(9) $$\;h_{s,t}=o_{t}\circ C_{t}\;$$

In these equations, $C_{t}$ denotes the hidden states, and ∘ stands for element-wise multiplication. σ is the sigmoid function, and *W_f_*, *b_f_*, *W_i_*, *b_i_*, *W_o_*, *b_o_*, *W_c_*, and *b_c_* are the parameters in the LSTM unit. Furthermore, we represent [*W_f_*, *W_i_*, *W_o_*, *W_c_*] as *W* and [*b_f_*, *b_i_*, *b_o_*, *b_c_*] as *B*.

During the entire training process, the main and auxiliary tasks share the relevant parameters (*W*, *B*) of the layer network, as shown in Formula (10):

(10) $$\{\begin{array}{c} h_{sk}=fun\left(x_{k},h_{s,k-1},C_{k-1},W,B\right)\\ h_{sk}^{\prime }=fun\left(y_{k},h_{s,k-1}^{\prime },C_{k-1}^{\prime },W,B\right) \end{array}$$

#### 3.2.3. Attention Mechanism

We can predict students’ performance while using the learning process and the overall learning state. In this section, we use the attention mechanism to adjust the weights of the learning process and the overall learning state to achieve better prediction.

Temporal attention focuses on the effects of temporal features, including temporal individual features and temporal grouping features, on students’ performance. The LSTM neural network is an improved neural network algorithm that can deal with temporal features for prediction, and solve the problems of gradient disappearance and gradient explosion.

Comprehensive attention focuses on the impact of students’ overall learning status on their performance. The existence of the forget gate causes the loss of some important information, although LSTM solves the problem of long-term dependence in feature sequences. We add an MLP neural network in Figure 1d using comprehensive features to supplement the prediction results that were obtained using temporal features to better reflect students’ behavior and predict students’ performance.

### 3.3. Loss Function with Cross Entropy 

#### 3.3.1. Loss Function

Cross-entropy is a concept in information theory [34,35,36,37,38] and it is used to express the similarity of two probability distributions. The smaller the value of cross-entropy is, the closer the two probability distributions are. We use cross-entropy as a loss function for training the neural network. In our method, we use the ensemble loss from the multitask multi-layer LSTM neural network and the MLP, as shown in Figure 1b,c, respectively. 

As shown in Formula (11), we use attention-based loss for the loss of MLP in Figure 1c. In this formula, *g* is the input of MLP, *w_g_* is the weight of the loss, *LM* is the cross entropy loss of MLP, and *GL* is the attentional loss of MLP.

(11) $$GL\left(\;\right)=w_{g}LM\left(g\right)$$

The Loss *LM*(*g*) can be calculated by Formula (12). *q* is the actual probability distribution for a sample and *p* is the output probability distribution from the MLP. *S* is number of samples and *x* is the position in the probability distribution.

(12) $$LM\left(g\right)=\overset{S}{\underset{s=1}{\sum }}\left(-\sum_{x}q\left(s,x\right)\log(p\left(s,x\right)\right))$$

For a sample *g* in the MLP neural network of *n* classification, we can transform the output [*z_1_*, *z_2_*,…, *z_n_*] into the probability distribution *p* while using the softmax function, as shown in Formulas (13) and (14). The actual classification of the sample can be transformed into the probability distribution *q* as *q =* [*0*, *0*... *1, 0... 0*], where 1 represents correct classification of the sample and 0 represents misclassification.

(13) $$p_{k}=softmax\left(z_{k}\right)=\frac{e^{z_{k}}}{\sum_{j=1}^{n}e^{z_{j}}}$$

(14) $$\;p=\left[\frac{e^{z_{1}}}{\sum_{j=1}^{n}e^{z_{j}}}\;,\;\;\frac{e^{z_{2}}}{\sum_{j=1}^{n}e^{z_{j}}}\;,\;\ldots \ldots \;,\;\frac{e^{z_{n}}}{\sum_{j=1}^{n}e^{z_{j}}}\;\;\;\right]\;$$

As shown in Formulas (15) and (16), we use attention-based loss of multi task in Figure 1b, where task loss (TL) is the ensemble loss of multiple tasks and *TL(c)* is the loss of task *c*.

(15) $$TL\left(c\right)=\overset{M}{\underset{k=1}{\sum }}w_{c}^{k}*LL\left(k,x_{c},h_{t}\right)$$

(16) $$\;w_{c}=\left[w_{c,}^{1},w_{c}^{2},w_{c}^{3}\ldots \ldots ,w_{c}^{M}\right]\;$$

Furthermore, $w_{c}$ is the weight of task *c*, M is the number of LSTM units in one layer, $w_{c}^{k}$ is the weight of *k*th unit in the last layer of task *c* for classification, *x_c_* is the input of the shared parameters layer in task *c*, and *LL*(*k*, *x_c_*, $h_{t}$) is the loss of *k*th unit in the last layer of LSTM. *LL* can be calculated similarly as Formula (12). 

We define an attention-based loss function to coalesce classification errors from all tasks. Comprehensive attention focuses on comprehensive features in the MLP model, while temporal attention focuses on temporal individual features and the temporal grouping features in the LSTM model. We adjust our attention by a mixed loss function and multiple weights.

For our method, the combined loss *CL* is shown as Formula (17):

(17) $$CL\left(\;\right)=GL\left(\;\right)+\overset{T}{\underset{c=1}{\sum }}\left(TL\left(c\right)\right)$$

where *T* is the number of tasks.

#### 3.3.2. Training

As shown in Figure 1d, the ensemble loss is composed of the losses of the two neural networks in Figure 1b and the losses of the neural networks in Figure 1c. We introduce the training process for parameters with ensemble cross entropy loss of the neural network while taking the MLP neural network as an example.

As described in the preceding section, [*z_1_*, *z_2_*, *…*, *z_n_*] is the output of the MLP neural network for a sample, *p* is the probability distribution that is outputted by the neural network, and *q* is the actual probability distribution. We first deduce the derivative of $p_{i}$ with respect to $z_{j}$, as shown by Formula (18) (refer to Formula (13)).

(18) $$\begin{array}{ccc} \frac{\partial p_{i}}{\partial z_{j}} & =\{\begin{array}{c} p_{i}-p_{i}*p_{j}\;\;\;i=j\\ -p_{i}*p_{j}\;\;\;\;\;\;i!=j \end{array} & \end{array}$$

Subsequently, we deduce the derivative of ensemble loss *CL* with respect to $z_{j}$. When we derive the output of a neural network with total loss, it is independent of the other neural networks. The process is shown in Formula (19).

(19) $$\begin{array}{ccc} \frac{\partial CL}{\partial z_{j}} & = & \frac{\partial GL}{\partial z_{j}}+\overset{T}{\underset{c=1}{\sum }}\frac{\partial TL\left(c\right)}{\partial z_{j}}\\ & = & \frac{\partial GL}{\partial z_{j}}\\ & = & w_{g}\overset{n}{\underset{i=1}{\sum }}\frac{\partial \left(-\sum_{x}q\left(x\right)\log(p\left(x\right)\right))}{\partial z_{j}}\\ & = & w_{g}\overset{n}{\underset{i=1}{\sum }}\frac{\partial \left(-\sum_{x}q\left(x\right)\log(p\left(x\right)\right))}{\partial p_{i}}*\;\frac{\partial p_{i}}{\partial z_{j}}\\ & = & -w_{g}\overset{n}{\underset{i=1,i!=j}{\sum }}\frac{q_{i}}{p_{i}}*\;\frac{\partial p_{i}}{\partial z_{j}}-w_{g}{\left(\frac{q_{i}}{p_{i}}*\;\frac{\partial p_{i}}{\partial z_{j}}\right)}_{i=j}\\ & = & -w_{g}\overset{n}{\underset{i=1,i!=j}{\sum }}\frac{q_{i}}{p_{i}}*\left(-p_{i}*p_{j}\right)-w_{g}\frac{q_{i}}{p_{i}}\left(p_{i}-p_{i}*p_{j}\right)\\ & = & -w_{g}\left(q_{i}-q_{i}*p_{j}\right)+w_{g}\overset{n}{\underset{i=1,i!=j}{\sum }}q_{i}*p_{j}\\ & = & w_{g}\left(p_{j}-q_{j}\right) \end{array}$$

After the above deduction, we can use the gradient descent method to reduce the overall loss and train all of the parameters. In addition, $w_{c},\;\;w_{g}$ can also be learned by minimizing the ensemble loss (CL) by training all the records. Experiments demonstrate that the share network for related tasks can improve the performance of the network. 

## 4. Experiment 

### 4.1. Data

For this study, we chose a C programming MOOC course offered by a university from February 2012 to June 2012. A total of 1528 students took the course. These students are sophomores and their average age is 19. This course is a compulsory course for all students, and the exam of this course is rather difficult, as not all students are expert in programming. The number of students who score more than 70 is 448 and who score more than 60 is 650 in the final exam. In this course, there were 69 programming assignments, and it lasted for 14 weeks. For each student, *X* and *Y* in Figure 1 are feature sequences of 14 lengths as the course lasts 14 weeks. The students were required to complete the assignments, and the grades for all of the assignments were converted into a portion of the final grade. The final grade for the course comprised the final exam and assignment grades. The data cannot be disclosed due to confidentiality agreement.

**Figure A1:**
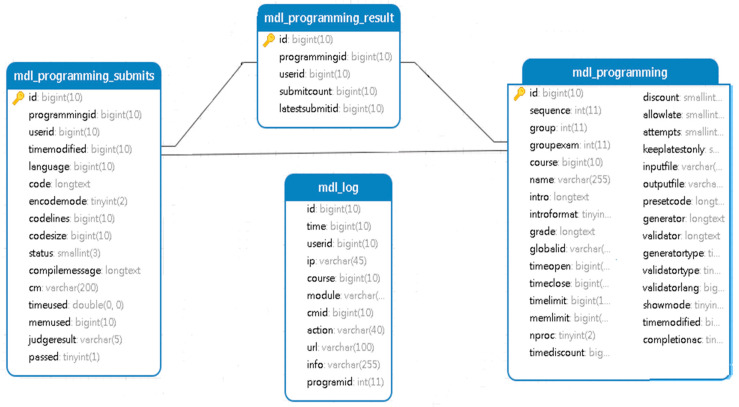
Logs from MOOC platform.

Table A1 represents the data from one programming assignment in which the students write a program that determines whether the three line segments can form a triangle. It is difficult to pass all the test cases by submitting the assignment only once, as several difficult hidden test cases exist in some complex assignments. We found that some of the students who had submitted and passed all of the test cases at once had copied the assignment from other students who had already completed it. Figure A1 shows some related log files to elucidate the structure of the log in the database. We extracted 34 features from the logs of the assignments; Table A2 lists a few of the features and their corresponding meanings.

**Figure 3:**
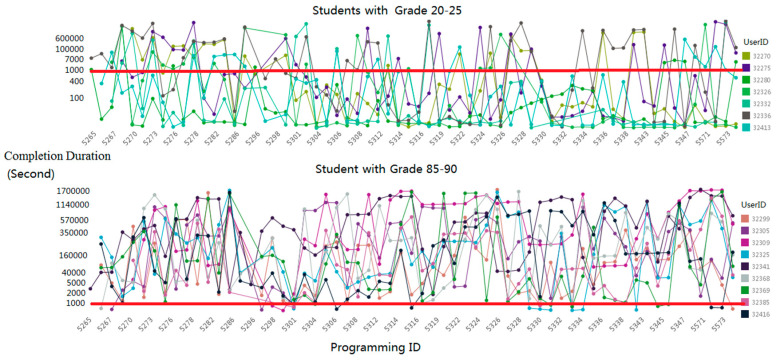
Relationship between completion duration and grade.

Figure 3 illustrates the relationship between the final grades and the completion duration. Students with grades between 85 and 90 rarely accomplish their assignments in 300 s, while students with grades between 20 and 25 complete several assignments in 300 s; this reveals that the completion of the assignments in a very short time period might indicate plagiarism. As programming assignments have special requirements for the output format, few students submit it after finishing their assignments in other places. 

**Figure 4:**
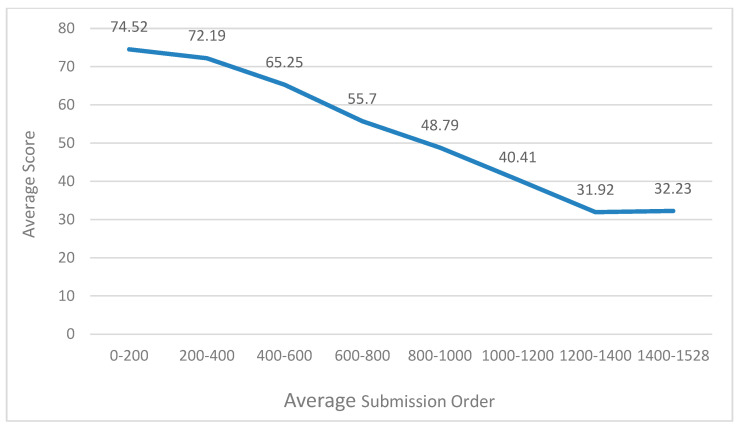
Relationship knowledge between average score and average submission order.

Figure 4 reveals the relationship between the average score and average submission order. Submission order refers to the order in which students submit their assignments. The submission order of the student who first submits his assignment is 1, and the submission order of the student who second submits his assignment is 2. Students with an average submission order between 0 and 200 for all assignments have an average score of 74.52, and students with an average submission order between 1400 and 1528 for all assignments have an average score of 32.23, as shown in Figure 4. Although the submission order does not completely determine students’ score, we can find that the submission order has a great relationship with students’ score. The average submission order reflects students’ learning initiative and motivation; students who submit earlier have higher learning initiative and may get a higher score.

We divided the assignment into groups according to the points and extracted the grouping features to predict the corresponding knowledge points’ mastery. We manually labeled the programming assignments and divided the programming assignments into 10 groups based on knowledge points. Table 1 summarizes the details. 

Of the 69 assignments, 13 were related to drawing a figure, 14 to a calculation, and eight to recursive questions. In the follow-up experiments, we paid attention to the mastery of these three knowledge points. We can predict students’ scores for the three knowledge points in the final examination while using students’ behavior in these 35 assignments of drawing a figure, calculation, and recursive questions. 

### 4.2. Baselines

We experimented with standard naïve Bayes (NB), standard logistic regression (LR), standard multi-layer perceptron (MLP), LSTM [28], M-F-LSTM [39], M-S-LSTM [40], and our method.

LSTM [28] is an improved recurrent neural network that can transmit information in a long sequence and solve the problem of gradient explosion and disappearance.

M-F-LSTM [39] is a fully-connected multi-layer LSTM among neural network layers. It has better performance in terms of delivering key information and forgetting unimportant information when compared to the standard LSTM neural networks.

M-S-LSTM [40] is a multi-task neural network with a shared layer. Different tasks share the same parameters in the shared layer; furthermore, it can mix multi-domain knowledge to improve the prediction accuracy and avoid over-fitting.

### 4.3. Evaluation Metrics 

We can predict their achievement and mastery of knowledge points while using the data of students’ behavior. For the prediction of achievement, we label their grades on a 70-point scale in the final exam. We selected three knowledge points with a total score of 30 in the final exam and labeled them with a threshold score of 21 for the prediction of knowledge points. We use behaviors related to all assignments to predict the student achievement and corresponding assignments to predict the mastery of knowledge point.

We use accuracy, recall, and cross-validation scores to evaluate the performance of the algorithm. In the prediction process, we use 80% of the data for training, and the remaining 20% for prediction. Furthermore, we use five-fold cross validation to calculate the cross validation score. We take the average value of the experimental results for five times, and use the variance estimation to measure the stability of the algorithm of the results.

### 4.4. Experimental Details 

We use the tensor flow framework and GPU for training the neural network. In the training process, there are 14 LSTM units in each layer for the main task, auxiliary task, and shared layers. For the main task and auxiliary task, the layer number is 3. We set *weight_decay* to 1e-5, *hidden_size* to 32, *drop_out* to 0.9, and *batch_size* to 256. After iterative training, we get the parameter values of each module, among which w_1_ = 0.571, w_2_ = 0.286, and w_3_ = 0.143. It means that the main task of temporal attention occupies the largest weight subsequent to training, the auxiliary task of temporal attention occupies the medium weight, and comprehensive attention occupies the smallest weight.

### 4.5. Result

In this subsection, we predict the performance of students while using temporal features as the main task separately and detect the mastery of knowledge points using grouping temporal features as the auxiliary task separately. We then test our multi-task framework while using individual temporal, grouping temporal, and comprehensive features. In the main task, we predict whether the student will pass the final exam or not; in the auxiliary task, we predict whether the student will attain a 60% score for the corresponding knowledge points. The experimental results are as follows: 

Table 2 lists the results that predict using features whether a student would pass the final exam:

We then separately predict the mastery of knowledge points using grouping temporal features. In this programming course, the programming tests in the final exam comprise a calculation, drawing figures, and recursive questions, with a top score of 30. We extract features from the logs of these labeled programming tests. We use these logs to predict the students’ programming grade in the final examination. Table 3 summarizes the results and indicates that we could use grouping temporal features to predict the performance of corresponding knowledge points; furthermore, we could find the weaknesses of the students.

**Figure 5:**
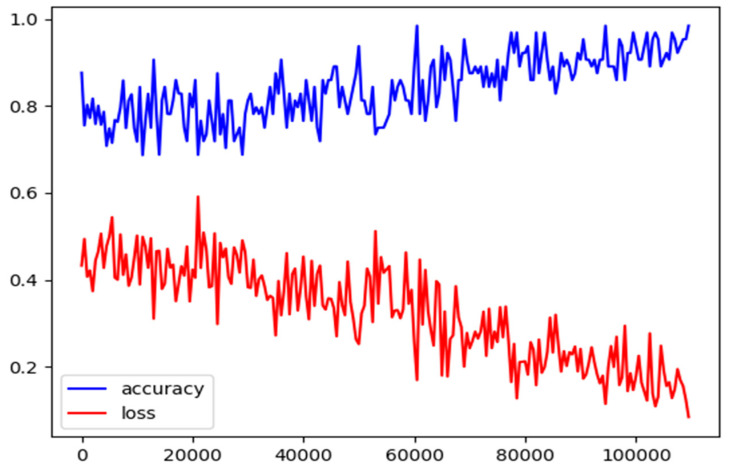
Variations of loss and accuracy of the proposed method with iterations.

We use the proposed method to predict both the main and auxiliary tasks by performing 100,000 iterations. Figure 5 shows the process of iteration. The losses converge after 100,000 iterations.

In our experiments, since LSTM and M-F-LSTM are single task framework, they only predict students’ performance and mastery of knowledge points, respectively. While our algorithm and M-S-LSTM are multi task algorithms, they predict students’ performance and mastery of knowledge points in the same framework. The results demonstrate that the proposed method can predict the students’ mastery of knowledge points and can achieve better performance when combined with the selected temporal features in the multi task framework, as shown in Table 4. Among these methods when predicting students’ mastery of knowledge points, the F1 Score of LSTM, M-F-LSTM, M-S-LSTM, and our method are 0.8956, 0.9792, 0.9912, and 0.9936, respectively, and the multi-task methods (M-S-LSTM and our method) have gained better performance in predicting students’ mastery of knowledge points. The ranges of LSTM, M-F-LSTM, M-S-LSTM, and our method at 95% confidence interval (CI) of F1 Score while predicting student performance are 0.0082, 0.0063, 0.0082, and 0.0031, respectively, and fully-connected LSTM based methods (M-F-LSTM and our method) have gained a lower range of the 95% CI of F1 Score. 

These results reveal that the multi task framework with different features can get good prediction results, and fully-connected LSTM based methods have low range of the CI.

## 5. Conclusions

In this study, we identified influential online assignment behaviors by building a classification model that can predict students’ final exam performance and mastery of each knowledge point in MOOCs. We presented a multi-task multi-layer LSTM-based student performance prediction method while using the cross-entropy loss function to predict the overall performance of students and their mastery of each knowledge point based on the selected features. A shared parameters layer is placed to avoid over-fitting and improve the accuracy of prediction. Subsequently, attention mechanism is used to adjust the weights of the learning process and the overall learning state to achieve better prediction. Our method performs better and achieves an accuracy of 92.52% in predicting students’ performance and a recall rate of 94.68% when compared with LSTM, M-F-LSTM, and M-S-LSTM. This work contributes to understanding the relationship between students’ online behavior and performance and it has the potential to maximize the effectiveness of tutors and help students in need. Another advantage of this work is that it can predict knowledge points that students will have trouble learning, which can enable tutors to provide more targeted help to students. In the future, we will do further research on the contents of assignments completed by students.

## Figures and Tables

**Table 1:** Programming assignments grouped by knowledge points.

Description	Number of Assignments
Basic knowledge	7
Drawing figure	13
Computational problems	14
Recursive structure	8
Case structure (if/else)	4
Loop structure (for and loop)	6
String operation	9
File operation	2
Sorting	2
Comprehensive calculation	4

**Table 2:** Separate predictions of performance.

	Precision	Recall
LR	0.729	0.7163
SVM	0.7452	0.7065
NB	0.7437	0.7366
MLP	0.7539	0.7536
LSTM	0.8019	0.9283

**Table 3:** Separate predictions of the mastery of knowledge points.

	Precision	Recall
LR	0.7218	0.7190
SVM	0.7338	0.6582
NB	0.7290	0.7222
MLP	0.7372	0.7333
LSTM	0.8201	0.9865

**Table 4:** The experimental results on student performance and mastery of knowledge point prediction.

Method		Precision	Recall	F1 Score (95% CI)
LSTM	Performance	0.8019	0.9283	0.8605(0.8564–0.8646)
	Knowledge Point	0.8201	0.9865	0.8956(0.8896–0.9017)
M-F-LSTM [39]	Performance	0.9126	0.9285	0.9205(0.9173–0.9236)
	Knowledge Point	0.9733	0.9852	0.9792(0.9771–0.9814)
M-S-LSTM [40]	Performance	0.9248	0.9456	0.9351(0.9310–0.9392)
	Knowledge Point	0.9928	0.9896	0.9912(0.9888–0.9935)
The proposed method	Performance	0.9252	0.9468	0.9359(0.9347–0.9371)
	Knowledge Point	0.9958	0.9914	0.9936(0.9920–0.9951)

## Appendix A

The assignment contains seven test cases. The first four are known to students, and the last three are hidden from them. After the student submits the assignment they have programmed, the MOOC platform will compile the assignment, enter the test inputs and judge whether the outputs of the students’ assignment are consistent with the expected outputs of the respective test cases. If it is consistent, the student will pass the test case.

**Questions:**

Give the length of three line segments and judge whether they can form a triangle.

**Input:**

The length of segment a, b and c.

**Output:**

Whether they can form a triangle.

Figure A1 shows some related log files, in which mdl_programming stores the contents of assignments including name, introduction, start time, end time, and other information. Mdl_programming_submits stores information regarding every assignment submitted by the students, including completion time (when students submit their assignment), content, compiled information, and test results. Mdl_programming_result stores the final result of the assignment for each student. Mdl_log stores students’ behavior on the MOOC platform including browsing assignments, watching videos, visiting time on each page, and so on.

We extracted 34 features from the logs of the assignments; a few features and their corresponding meanings are listed in Table A2. Owing to the particularity of programming assignments, to ensure that the programming assignment can pass all the test cases, students may submit the programming assignment several times. Submission times is the number of times that the students submit their assignments. Completion time refers to the time when a student has submitted an assignment. The submission order refers to the ranking of one student’s completion time in relation to that of all other students. Completion duration is time between when a student first views the assignment to when they finally submit it. If a student’s completion duration for an assignment is under 300 s, it is considered as a quick submission. As it is almost impossible to complete the assignments within 300 s owing to the difficulty of the assignments, quick submission means that the student is likely to have copied the assignment from others. The number of quick submissions by students reflects the degree of their cheating.

**Table A1:** Test Cases.

ID	Public	Test Input	Test Output	Time Limitation	Memory Limitation	Weight
1	Yes	3, 4, 5	Yes	1 s	0KB	1
2	Yes	1, 4, 6	No	1 s	0KB	1
3	Yes	−1, 2, 3	Error Input	1 s	0KB	1
4	Yes	3, 6, 6	Yes	1 s	0KB	1
5	No	3, 3, 3	Yes	1 s	0KB	1
6	No	−5, 7, 9	Error Input	1 s	0KB	1
7	No	1, 4, 9	No	1 s	0KB	1

**Table A2:** Features.

Feature	Meaning
Average First Submission Order	Order of submitting one assignment to the website for the first-time
Average Submission Times	Average value of submission times for all assignments
Average Completion Duration	Average value of completion duration for all assignments
One-Time Pass Number	Number passing all cases for one submission in all assignments
Quick Submission Times	Quick submission times of all assignments
Completion time	The time when a student submitted an assignment
